# Supplementary material for: Programmed Double-stranded RNA Formation Enables Meiotic Stage Transitions
Source: bioRxiv. 2026 Jan 20:2026.01.20.700650. Preprint. [Version 1] doi: 10.64898/2026.01.20.700650 (PMC12871743; doi:10.64898/2026.01.20.700650)
Supplement: 1 [file NIHPP2026.01.20.700650V1-supplement-1.pdf]

bioRxiv preprint doi: https://doi.org/10.64898/2026.01.20.700650; this version posted January 20, 2026. The copyright holder for this preprint (which was not certified by peer review) is the author/funder, who has granted bioRxiv a license to display the preprint in perpetuity. It is made available under aCC-BY 4.0 International license.

SUPPLEMENTAL FIGURE TITLES AND LEGENDS

Figure S1. Time course of synchronized yeast meiosis and analyses on DMS reactivity changes across stages, related to Figure 1

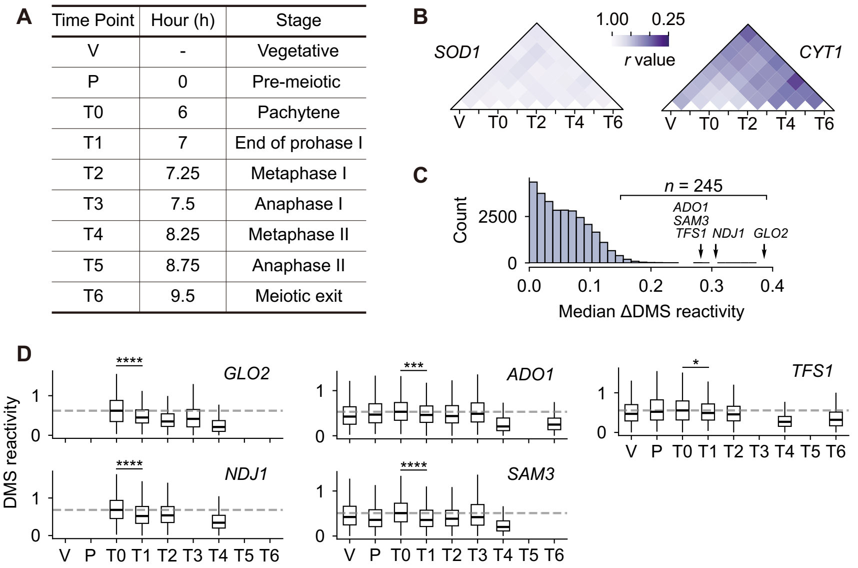

(A) Meiotic time points and corresponding stages in the synchronized Ndt80-inducible system. Hours represent time in SPO medium after Ndt80 induction at T0 (6 h). Staging is as defined in our previous study[21].

(B) Examples of conserved (*SOD1*) and strongly remodeled (*CYT1*) mRNA structures during meiosis.

(C) Distribution of median ΔDMS reactivities for 28,601 pairwise comparisons.

(D) Distribution of DMS reactivity at each stage for *GLO2*, *NDJ1*, *ADO1*, *SAM3*, and *TFS1*. For all five genes, DMS reactivity drops significantly between T0 and T1 and reaches its maximal decrease by T4 (median change shown). The dashed line indicates the median DMS reactivity at T0.

bioRxiv preprint doi: https://doi.org/10.64898/2026.01.20.700650; this version posted January 20, 2026. The copyright holder for this preprint (which was not certified by peer review) is the author/funder, who has granted bioRxiv a license to display the preprint in perpetuity. It is made available under aCC-BY 4.0 International license.

Figure S2. Analyses on antisense read ratios and validation of antisense RNAs by northern blot, related to Figure 2

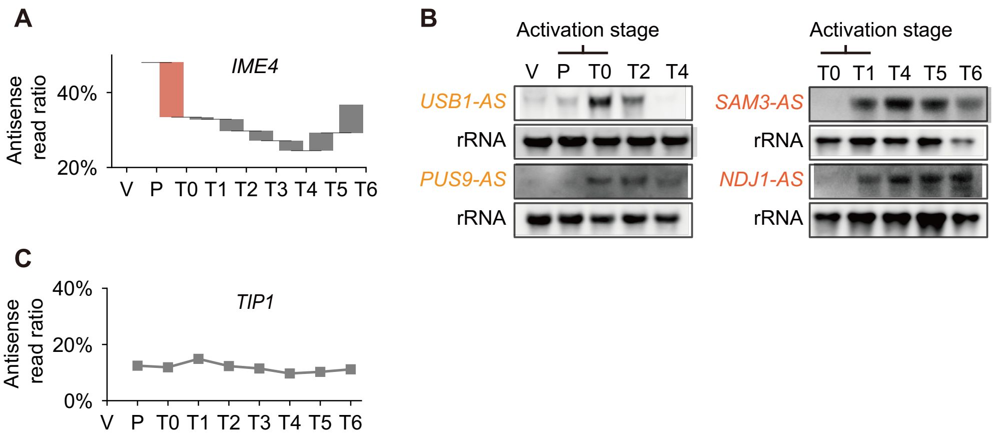

(A) Antisense read ratio of *IME4* throughout meiosis. The orange segment highlights the interval with the largest change.
(B) Northern blot analysis of antisense RNAs: *USB1-AS*, *PUS9-AS*, *SAM3-AS*, and *NDJ1-AS*. The activation stage is defined as the interval with the largest increase in antisense read ratio.
(C) Antisense read ratio of *TIP1* throughout meiosis. The ratio remains stable at a background level of 15%–20%.

bioRxiv preprint doi: https://doi.org/10.64898/2026.01.20.700650; this version posted January 20, 2026. The copyright holder for this preprint (which was not certified by peer review) is the author/funder, who has granted bioRxiv a license to display the preprint in perpetuity. It is made available under aCC-BY 4.0 International license.

Figure S3. Motif enrichment analysis results and overview of 5′ end mapping by long-read sequencing, related to Figure 3

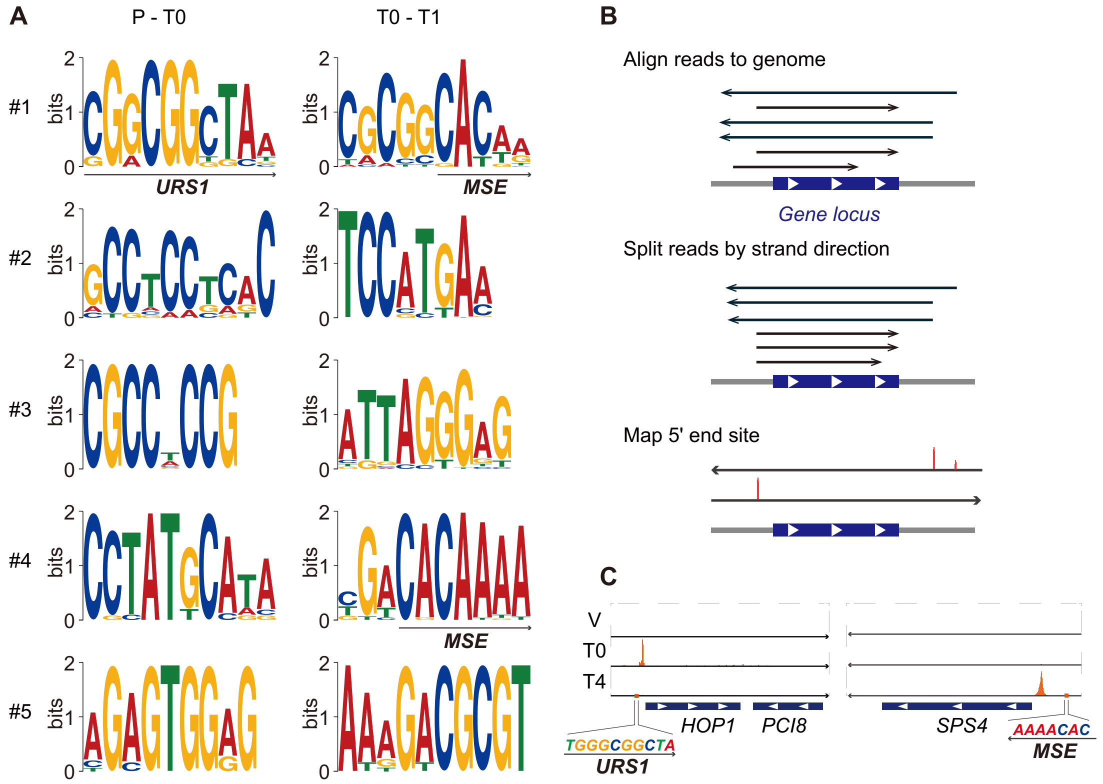

(A) Top five motifs enriched in the motif enrichment analyses for antisense promoters activated in the P-T0 and T0-T1 intervals. Core sequences of the URS1 (Ume6-binding) and MSE (Ndt80-binding) elements are highlighted within the respective motifs.
(B) Overview of long-read sequencing and identification of transcription initiation sites based on 5′ end mapping. Transcription initiation sites are indicated by the orange peaks.
(C) Transcription initiation sites (orange peak) for *HOP1* and *SPS4* sense mRNAs at the V, T0, and T4 stages.

bioRxiv preprint doi: https://doi.org/10.64898/2026.01.20.700650; this version posted January 20, 2026. The copyright holder for this preprint (which was not certified by peer review) is the author/funder, who has granted bioRxiv a license to display the preprint in perpetuity. It is made available under aCC-BY 4.0 International license.

Figure S4. Sense mRNA profiles, Rny1p abundance across meiosis and *AKR2* mRNA localization analysis, related to Figure 4

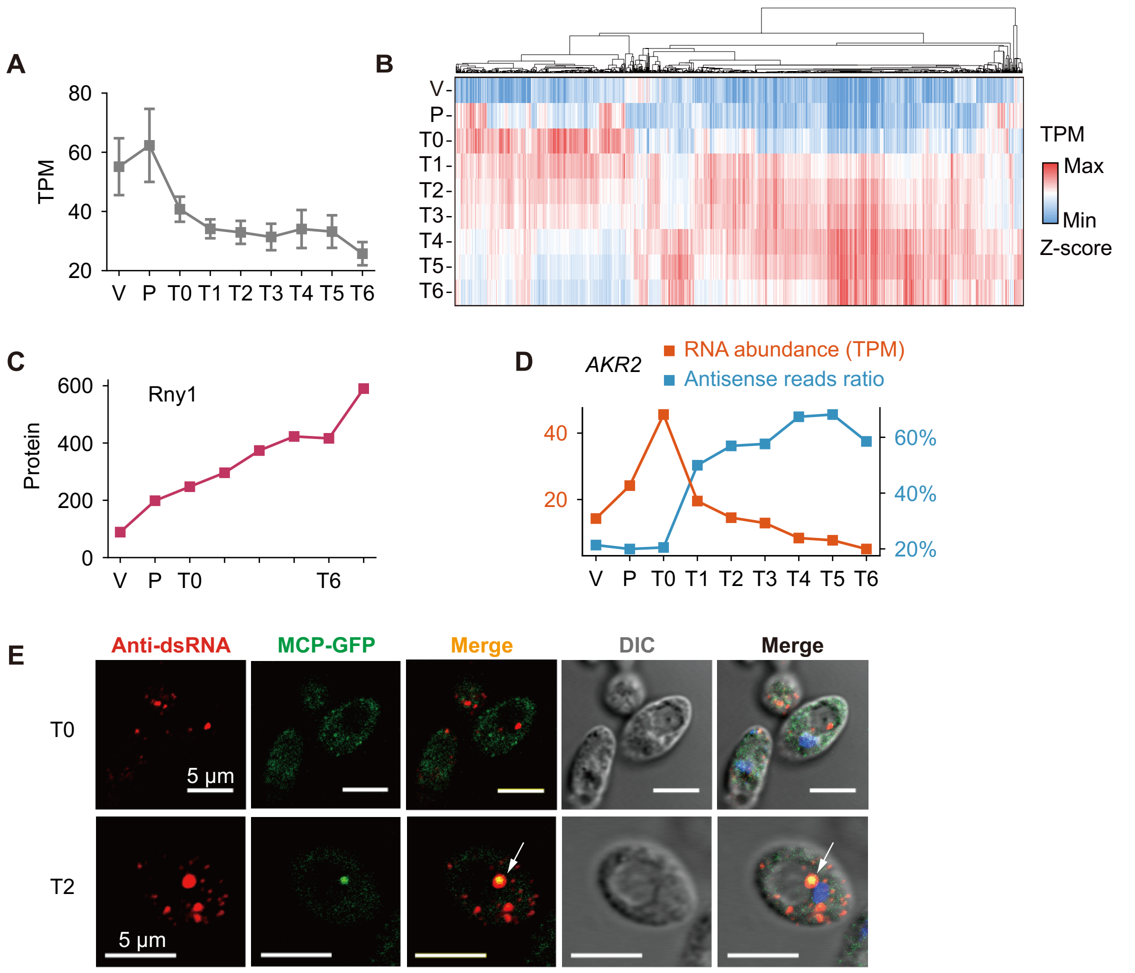

(A) Average RNA abundance of 313 genes exhibiting meiosis-specific antisense transcription.
(B) Hierarchical clustering of RNA abundance for 1,044 transcripts that were upregulated more than eightfold during meiosis and did not exhibit meiosis-specific antisense transcription.
(C) Protein abundance of Rny1 throughout meiosis detected by mass spectrometry[28].
(D) Relationship between *AKR2* RNA abundance and antisense read ratio.
(E) Co-localization analysis of *NDJ1-MS2* with dsRNA aggregates before (T0) and after (T2) Ndt80 induction. White arrows indicate co-localization between dsRNA signals and MCP-GFP.

bioRxiv preprint doi: https://doi.org/10.64898/2026.01.20.700650; this version posted January 20, 2026. The copyright holder for this preprint (which was not certified by peer review) is the author/funder, who has granted bioRxiv a license to display the preprint in perpetuity. It is made available under aCC-BY 4.0 International license.

Supplemental information

Table S1. Pearson correlation analysis of DMS reactivities and median ΔDMS reactivity for 28,601 pairwise comparisons across 2,058 mRNAs, related to Fig. 1A and 1B.

Table S2. Antisense read ratios for 5,520 gene loci across all time points. Genes with changes greater than 20% and no more than one missing value were used for hierarchical clustering, related to Fig. 2A.

Table S3. Genes with meiosis-specific NATs (n = 313) classified by meiotic stage of maximum increase in antisense read ratio, related to Fig. 2B and 2C.

Table S4. Motif enrichment analysis results for antisense promoters of genes classified into P-T0 and T0-T1 intervals, related to Fig. 3A and S3A.

Table S5. Raw results of RT-qPCR for MSE deletion experiments, related to Fig. 3E.

Table S6. Hierarchical clustering results of sense mRNA abundance profiles for genes associated with meiosis-specific NATs, related to Fig. 4A.

Table S7. Peak calling results for Ume6p and Ndt80p binding sites, related to Fig. 4E. The ChIP-seq data used in this analysis are from previously published datasets[29,30].
